# Decrease in Intracellular Perforin Levels and IFN-*γ* Production in Human CD8^+^ T Cell Line following Long-Term Exposure to Asbestos Fibers

**DOI:** 10.1155/2018/4391731

**Published:** 2018-10-23

**Authors:** Naoko Kumagai-Takei, Yasumitsu Nishimura, Hidenori Matsuzaki, Suni Lee, Kei Yoshitome, Takemi Otsuki

**Affiliations:** ^1^Department of Hygiene, Kawasaki Medical School, 701-0192 Kurashiki, Japan; ^2^Department of Life Science, Faculty of Life and Environmental Science, Prefectural University of Hiroshima, 727-0023 Shobara, Japan

## Abstract

Although the tumorigenicity of asbestos, which is thought to cause mesothelioma, has been clarified, its effect on antitumor immunity requires further investigation. We previously reported a decrease in the percentage of perforin^+^ cells of stimulated CD8^+^ lymphocytes derived from patients with malignant mesothelioma. Therefore, we examined the effects of long-term exposure to asbestos on CD8^+^ T cell functions by comparing long-term cultures of the human CD8^+^ T cell line EBT-8 with and without exposure to chrysotile (CH) asbestos as an *in vitro* model. Exposure to CH asbestos at 5 *μ*g/ml or 30 *μ*g/ml did not result in a decrease in intracellular granzyme B in EBT-8 cells. In contrast, the percentage of perforin^+^ cells decreased at both doses of CH exposure. CH exposure at 30 *μ*g/ml did not suppress degranulation following stimulation with antibodies to CD3. Secreted production of IFN-*γ* stimulated via CD3 decreased by CH exposure at 30 *μ*g/ml, although the percentage of IFN-*γ*^+^ cells induced by PMA/ionomycin did not decrease. These results indicate that long-term exposure to asbestos can potentially suppress perforin levels and the production of IFN-*γ* in human CD8^+^ T cells.

## 1. Introduction

The association between mesothelioma and asbestos exposure is undisputed [1, 2]. Previous studies have focused largely on the properties of asbestos fibers that are important in the development of MM and the mechanisms of action of asbestos in the multistage carcinogenic process [3–6]. However, it seems to be insufficient to exert all effort on studies concerning its carcinogenicity. In fact, the induction of malignant mesothelioma (MM) by exposure to asbestos is not a rapid process and takes a long period to develop [7–9]. This suggests the possibility that the development of MM might be related to other functional alterations and prompted us to suppose that exposure to inhaled asbestos might gradually impair the immune response.

On the basis of this hypothesis, we previously examined the effect of long-term exposure to asbestos on human NK and CD4^+^ T cell lines. The human NK cell line YT-A1 was cultured with continuous exposure to chrysotile (CH) asbestos at 5 *μ*g/ml, subsequently referred to as the YT-CB5 subline. YT-CB5 showed impaired cytotoxicity and decreased cell surface expression of several NK cell-activating receptors after around 5 months from the start of the culture [10]. For an *in vitro* model representing continuous exposure to asbestos in a human CD4^+^ T cell line, MT-2 cells were subjected to long-term exposure to asbestos and showed a reduction in cell surface CXCR3 expression [11]. Those immune-suppressive characteristics of asbestos exposure were also demonstrated by experiments with human primary cells, in which NK cells showed low expression of activating receptor NKp46 while CD4^+^ T cells showed low CXCR3. Additionally, suppressed expression of surface NKp46 and CXCR3 was also observed in NK cells and CD4^+^ T cells derived from peripheral blood of patients with MM [10, 12].

Our interest in the immunological effects of asbestos exposure also encompasses events that occur within CD8^+^ T cells. In antitumor immunity, CD8^+^ T cells as well as NK cells play a role as effectors which kill tumor cells [13]. Following conjugation of CD8^+^ T cells with an appropriate target cell, lytic granules are transported to the point of contact with the target, and granule perforin and granzyme B are released into the immune synapse between CD8^+^ T cells and the target. Once the granule contents are released by CD8^+^ T cells, they act on the target cell to induce apoptosis [14]. The granule core is surrounded by a lipid bilayer containing lysosome-associated membrane glycoprotein CD107a, which is known to be transiently expressed on the T cell surface during degranulation [15]. Recently, we also reported that patients with MM showed lower levels of perforin in CD8^+^ lymphocytes after stimulation compared to patients with pleural plaque (PP), and the percentage of IFN-*γ*^+^ cells in CD8^+^ lymphocytes of patients with MM tended to be lower compared to healthy volunteers (HV) and patients with PP [16].

The findings obtained from our aforementioned studies demonstrated the asbestos-induced functional impairment of immune cells by employing experiments using long-term cultures of cell lines and short-term cultures of primary cells with asbestos exposure. However, long-term exposure of a cultured CD8^+^ T cell line to asbestos has yet to be investigated. Therefore, we hypothesized that chronic and direct exposure to asbestos might affect the antitumor immunity of CD8^+^ T cells and examined this possibility by comparing long-term cultures of the human CD8^+^ T cell line EBT-8 with and without exposure to CH asbestos as an *in vitro* model to analyze the effects of asbestos exposure on CD8^+^ T cells. The human CD8^+^ T cell line EBT-8 was continuously cultured with asbestos and assayed for intracellular levels of perforin, granzyme B, and IFN-*γ* and degranulation and production of INF-*γ*.

## 2. Materials and Methods

### 2.1. Cell Culture of the Human CD8^+^ T Cell Line

The human CD8^+^ T cell line EBT-8 was a gift from Prof. H. Asada [17]. EBT-8 cell line was established from large granular lymphocyte leukemia of T cell origin and shows surface expressions of CD2, CD3, CD8, HLA-DR, and T cell receptor alpha/beta, which are characteristics of cytotoxic T lymphocytes. EBT-8 was established simply by continuously culturing mononuclear cells obtained from the patient with leukemia, which means EBT-8 was not established as a specific T cell clone for a specific antigen. Cells were cultured in GIT medium (Wako Pure Chemical Industries Ltd., Osaka, Japan) supplemented with 80 U/ml recombinant human IL-2 (Takeda, Osaka, Japan), 100 *μ*g/ml streptomycin, and 100 U/ml penicillin (Meiji Seika Pharma Co. Ltd., Tokyo, Japan). Cells were placed in 25 cm^2^ tissue culture flasks in portrait style in a volume of 10 ml and incubated at 37°C in a humidified atmosphere of 5% carbon dioxide in air. As an *in vitro* model, long-term cultures of the human CD8^+^ T cell line EBT-8 with and without exposure to chrysotile asbestos were performed. The International Union Against Cancer (UICC) standard sample of chrysotile A and B asbestos was kindly provided by the Department of Occupational Health at the National Institute for Occupational Health of South Africa [18]. A Japan Association for the Study of Fiber Materials (JASFM) standard sample of chrysotile asbestos, JAWE 131, was also used since the entire UICC standard sample of chrysotile asbestos was utilized. Chrysotile fibers were separated in PBS by pipetting repeatedly and used for culture. Most of the fibers were dispersed separately in the solution but included some part of bundle or cluster of fibers. The EBT-8 cell line was continuously cultured with chrysotile asbestos at 0, 5, or 30 *μ*g/ml for more than 1 month but less than 2 months in culture flask. Cells were regularly separated from asbestos using a Ficoll-Hypaque density gradient (Separate-L^®^, Muto Pure Chemicals Co. Ltd., Tokyo, Japan) before being used for functional assays.

### 2.2. Cell Growth Assay

EBT-8 cells (2.5 × 10^3^ cells per well in 96-well flat-bottomed plates) were cultured in 100 *μ*l of medium containing 0, 1, 5, 10, 20, 50, or 100 *μ*g/ml of asbestos. Following 48 h of culture, the growth properties were analyzed with a water-soluble tetrazolium salt (WST-1) assay using a Premix WST-1 Cell Proliferation Assay System (Takara Bio Inc., Shiga, Japan) according to the manufacturer's instructions. The wavelength for measuring the absorbance of the formazan product was 450 nm. The reference wavelength was 650 nm.

### 2.3. Assay for Expression Levels of Intracellular Molecules

To examine the expression levels of intracellular granzyme B and perforin, EBT-8-Org, EBT-8-CH5, and EBT-8-CH30 cells were fixed with 3.7% formaldehyde and then permeabilized with 0.1% Triton X-100. Fixed cells were then stained using the following antibodies (Abs): granzyme B-RPE, IgG1 negative control: RPE (both AbD Serotec, Oxford, UK), perforin-RPE, or IgG2b-RPE (both Ancell Corporation, Bayport, MN) Abs. For the staining of intracellular IFN-*γ*, 1.0 × 10^5^ EBT-8-Org or EBT-8-CH5 cells were stimulated with 50 ng/ml PMA and 250 ng/ml ionomycin (Sigma-Aldrich, St. Louis, MO) in GIT medium supplemented with 80 U/ml recombinant human IL-2, 100 *μ*g/ml streptomycin, and 100 U/ml penicillin in 96-well flat-bottomed plates. GolgiStop Protein Transporter inhibitor (containing monensin) (BD Biosciences, San Jose, CA) was also added to the medium for the staining of IFN-*γ*. Plates were incubated at 37°C for 4 h in a humidified atmosphere of 5% CO_2_. Stimulated EBT-8-Org or EBT-8-CH5 cells were harvested and stained with IFN-*γ*-PE Ab (BD Biosciences) using a Cytofix/Cytoperm^™^ Fixation/Permeabilization kit (BD Biosciences). The percentage or mean fluorescence intensity (MFI) of cells positive for each parameter was analyzed using a FACSCalibur^™^ (BD Biosciences) flow cytometer.

### 2.4. Detection of Degranulation

Cells were stimulated with bead-bound antibodies and assayed for degranulation. Monoclonal antibody CD3 (Beckman Coulter Inc., Brea, CA) and mouse IgG1 (BD Biosciences) were each incubated with anti-mouse IgG-coated beads (Spherotech Inc., Lake Forest, IL) at room temperature for 30 min. After washing the beads with PBS, 1.0 × 10^4^ cells were incubated with 1.0 × 10^4^ beads in GIT medium supplemented with 80 U/ml recombinant human IL-2, 100 *μ*g/ml streptomycin, and 100 U/ml penicillin in 96-well round-bottomed plates. After the plates were incubated at 37°C for 2 h in a humidified atmosphere of 5% CO_2_, cells were washed and stained with CD107a-PE Ab (BD Biosciences) before the percentage of CD107a^+^ cells was measured by flow cytometry.

### 2.5. Enzyme-Linked Immunosorbent Assay (ELISA)

Cells were stimulated by bead-bound antibodies in the same manner as described in Detection of Degranulation. After 48 hours of stimulation with anti-CD3-coated beads, culture supernatants of EBT-8-Org or EBT-8-J30 were collected and assayed for IFN-*γ*. The assay was performed using a Quantikine ELISA kit (R&D Systems Inc. Minneapolis, MN) according to the manufacturer's instructions.

### 2.6. Statistical Analysis

Significant differences (*p* < 0.05) were determined using analysis of variance with the post hoc test of Student-Newman-Keuls or paired Student's *t*-test.

## 3. Results

### 3.1. The Effect of Asbestos Exposure on Cell Growth of a Human CD8^+^ T Cell Line

**Figure 1:**
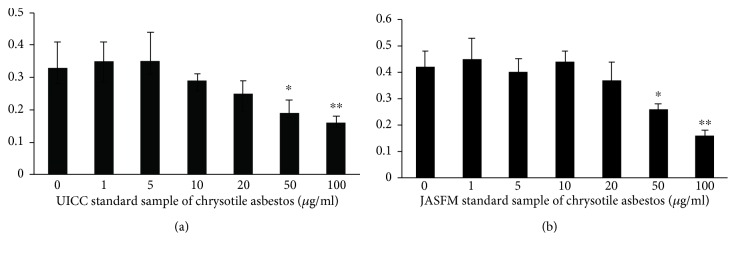
Cell growth of EBT-8 cells upon exposure to chrysotile asbestos. After EBT-8 cells were cultured with 0, 1, 5, 10, 20, 50, or 100 mg/ml of UICC (a) or JASFM (b) standard sample of chrysotile asbestos for 2 days, the WST-1 assay was performed and the absorbance was determined at the respective wavelength using a microplate reader. Data (a and b) represent the mean + SD from four wells. Significant differences are indicated by asterisks (^∗^*p* < 0.05, ^∗∗^*p* < 0.01).

To examine the effects of exposure to asbestos during a short period, the dose-dependent effects of exposure to CH asbestos on the growth of EBT-8 were assayed. The growth of EBT-8 cells was inhibited by exposure to a UICC standard sample of chrysotile asbestos at over 50 *μ*g/ml (Figure 1(a)). The growth of EBT-8 cells was also inhibited by exposure to a JASFM standard sample of chrysotile asbestos at over 50 *μ*g/ml (Figure 1(b)). EBT-8 cells were then cultured in the absence or presence of CH asbestos at low or middle concentrations of 5 or 30 *μ*g/ml for more than 1 month but less than 2 months and referred to as EBT-8-Org, EBT-8-CH5, and EBT-8-CH30, respectively.

### 3.2. Expression Levels of Granzyme B and Perforin following Long-Term Exposure to Low-Dose Asbestos

**Figure 2:**
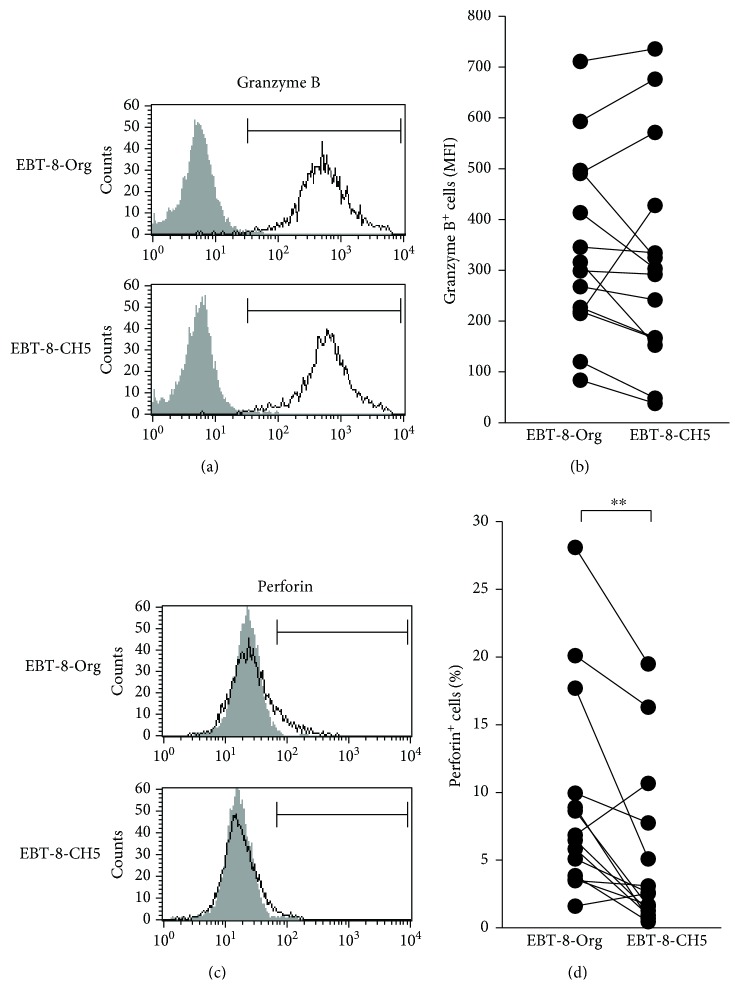
Expression levels of intracellular granzyme B and perforin following long-term exposure to low-dose asbestos. (a) Representative histograms of intracellular granzyme B in EBT-8-Org and EBT-8-CH5. An isotype control (gray) is shown in each panel. (b) Cumulative data showing the MFI of granzyme B-positive cells in EBT-8-Org or EBT-8-CH5. (c) Representative histograms of intracellular perforin in EBT-8-Org and EBT-8-CH5. An isotype control (gray) is shown in each panel. (d) Cumulative data showing the percentage of perforin-positive cells in EBT-8-Org or EBT-8-CH5. (b and d) Data represent values from 14 independent experiments. Significant differences are indicated by asterisks (^∗∗^*p* < 0.01).

To examine the effect of long-term exposure to low-dose asbestos on the functional properties of human CD8^+^ T cells, the expression levels of intracellular granzyme B in EBT-8-Org and EBT-8-CH5 were analyzed. As shown in Figures 2(a) and 2(b), the intracellular levels of granzyme B were very high in both EBT-8-Org and EBT-8-CH5 sublines with no significant difference in these levels between the sublines. As shown in Figures 2(c) and 2(d), the EBT-8-CH5 subline showed a statistically significant decrease in the percentage of perforin^+^ cells compared to EBT-8-Org. These results indicate that long-term exposure to CH asbestos at 5 *μ*g/ml can potentially cause a preferential decrease in intracellular perforin levels in CD8^+^ T cells while not affecting granzyme B levels.

### 3.3. Expression Levels of Granzyme B and Perforin following Long-Term Exposure to Middle-Dose Asbestos

**Figure 3:**
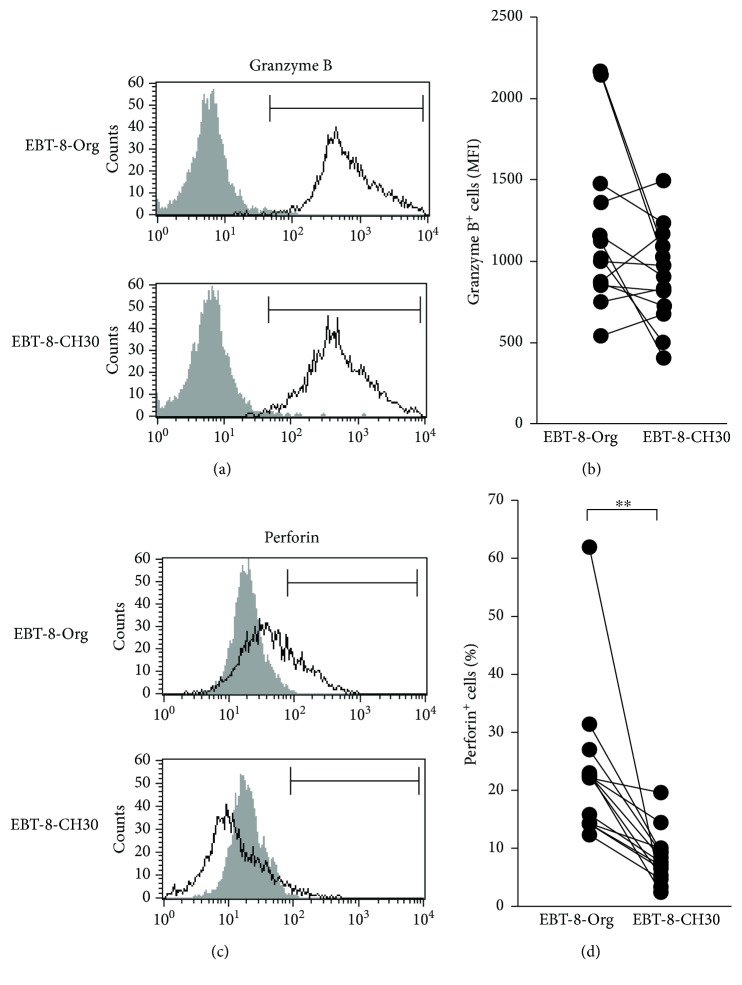
Expression levels of intracellular granzyme B and perforin following long-term exposure to middle-dose asbestos. (a) Representative histograms of intracellular granzyme B in EBT-8-Org and EBT-8-CH30. An isotype control is shown in each panel. (b) Cumulative data showing the MFI of granzyme B-positive cells in EBT-8-Org or EBT-8-CH30. (c) Representative histograms of intracellular perforin in EBT-8-Org and EBT-8-CH30. An isotype control (gray) is shown in each panel. (d) Cumulative data showing the percentage of perforin-positive cells in EBT-8-Org or EBT-8-CH30. (b and d) Data represent values from 14 independent experiments. Significant differences are indicated by asterisks (^∗∗^*p* < 0.01).

To examine the effect of long-term exposure to middle-dose asbestos on the functional properties of human CD8^+^ T cells, we continuously cultured EBT-8 cells with chrysotile asbestos at 30 *μ*g/ml in the same manner as described for the experiment utilizing low doses of asbestos. As shown in Figures 3(a) and 3(b), the expression level of granzyme B was very high in EBT-8-CH30, which was cultured at 30 *μ*g/ml, as well as in the Org subline, with no significant difference between the sublines. The EBT-8-CH30 subline showed a decrease in the percentage of perforin^+^ cells, with a significant difference compared to EBT-8-Org (Figures 3(c) and 3(d)). These results indicate that long-term exposure to CH asbestos even at 30 *μ*g/ml causes a preferential decrease in intracellular perforin levels in CD8^+^ T cells but does not alter the levels of granzyme B, in a similar manner to the low dose of 5 *μ*g/ml CH.

### 3.4. Degranulation Induced by TCR Stimulation following Long-Term Exposure to Asbestos

**Figure 4:**
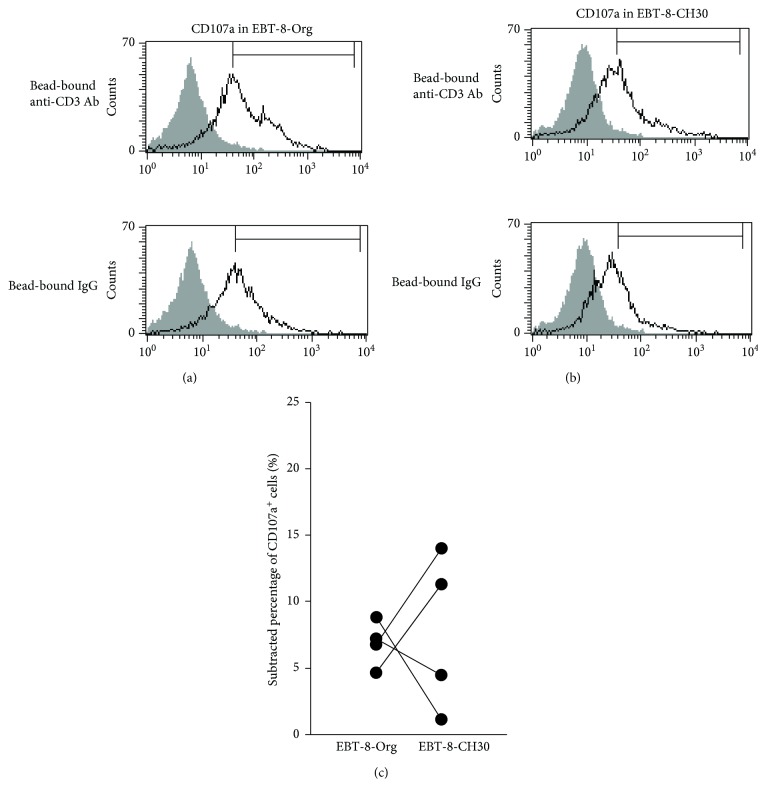
Degranulation induced by TCR stimulation following long-term exposure to asbestos. (a and b) Representative histograms of cell surface CD107a in EBT-8-Org (a) or EBT-8-CH30 (b) by stimulation with bead-bound anti-CD3 Ab or bead-bound IgG. A nonstained control (gray) is shown in each panel. (c) Cumulative data showing the subtracted percentage of CD107a^+^ cells in EBT-8-Org or EBT-8-CH30. Data represent values from 4 independent experiments.

Degranulation was compared between EBT-8-Org and EBT-8-CH30 since the degranulation of cytotoxic granules is a necessary step for the achievement of granzyme B/perforin-mediated cytotoxicity. To examine degranulation, the percentage of CD107a^+^ cells in stimulated cells with bead-bound IgG was subtracted from the percentage of CD107a^+^ cells in stimulated cells with bead-bound anti-CD3 Ab. EBT-8-Org and EBT-8-CH30 showed increases in cell surface expression of CD107a following stimulation with bead-bound Ab to CD3, although there was no significant difference between these sublines (Figures 4(a) and 4(b)). These results indicate that degranulation was not altered following exposure to CH asbestos, in contrast to the intragranular content of perforin.

### 3.5. Production of IFN-*γ* following Long-Term Exposure to Asbestos

**Figure 5:**
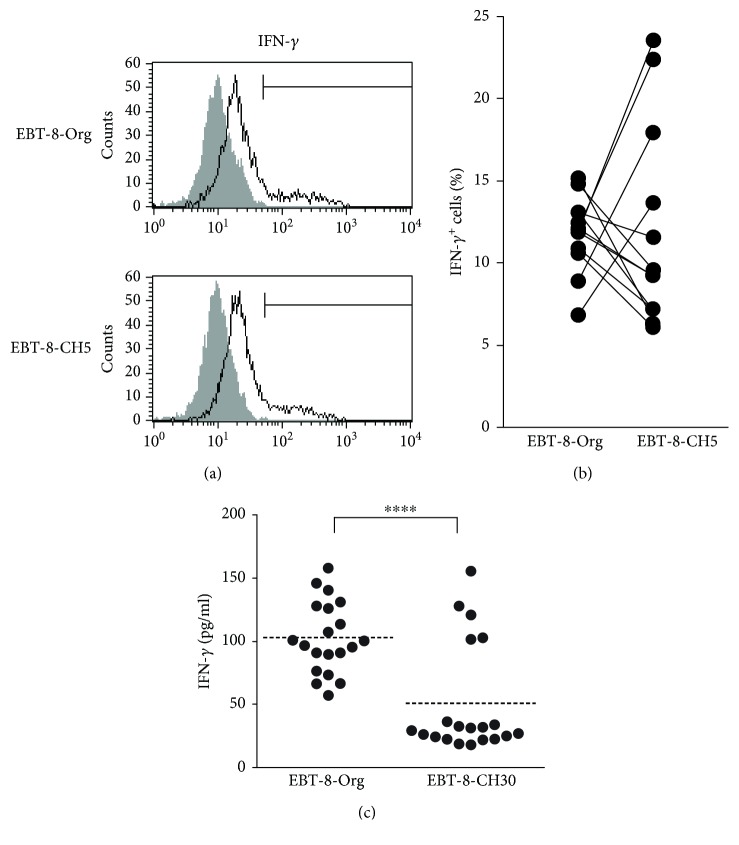
Production of IFN-*γ* following long-term exposure to asbestos. (a) Representative histograms of intracellular IFN-*γ* in EBT-8-Org or EBT-8-CH5. A nonstained control (gray) is shown in each panel. (b) Cumulative data showing the percentage of IFN-*γ*-positive cells in EBT-8-Org or EBT-8-CH5. Data represent values from 11 independent experiments. (c) Production of IFN-*γ* in culture supernatants of EBT-8-Org and EBT-8-CH30. Data represent values from 20 independent experiments. Horizontal dotted bars indicate the mean values. Significant differences are indicated by asterisks (^∗∗∗∗^*p* < 0.0001).

CD8^+^ T cells have been shown to be crucial for the immune response against tumors through the production of effector molecules including IFN-*γ* as well as granzyme B and perforin [19]. In an effort to examine the expression levels of IFN-*γ*^+^ cells in EBT-8-Org and EBT-8-CH5, the same sublines shown in Figure 2, these sublines were stimulated with PMA/ionomycin for 4 h (Figure 5(a)). As shown in Figure 5(b), exposure to low-dose CH asbestos did not suppress the percentage of IFN-*γ*^+^ cells. Next, we employed stimulation with bead-bound anti-CD3 Abs to represent greater physiological stimulation and examined the effect of middle-dose asbestos on the production of IFN-*γ* in the supernatants harvested following 48 hours of stimulation. The production of IFN-*γ* in culture supernatants of EBT-8-CH30 was clearly low, with significant difference compared to EBT-8-Org (Figure 5(c)). These results indicate that long-term exposure to CH asbestos at 30 *μ*g/ml suppresses the secretory production of IFN-*γ* by CD8^+^ T cells.

## 4. Discussion

EBT-8-CH30 showed decreased intracellular levels of perforin and production of IFN-*γ*. These results indicate that *in vitro* exposure to asbestos for long periods can potentially impair the functions of CD8^+^ T cells. Although these findings were obtained from *in vitro* experiments, it is possible that lymphocytes could be exposed to asbestos in the body. Inhaled asbestos fibers migrate to the pleural cavity and are thought to translocate into lymphatics which is open on the surface of parietal pleura [20]. Furthermore, it has also been reported that asbestos fibers accumulate in the lungs as well as regional lymph nodes in people exposed to asbestos occupationally as well as nonoccupationally [21, 22]. Therefore, our present demonstration using cell lines exposed to asbestos might provide a feasible model for examining the effect of chronic asbestos exposure on cytotoxic T lymphocytes in the body. The impaired function of CD8^+^ T cells might provide an opportunity for asbestos-induced abnormal cells which temporally arise with some features of DNA damage or transformation to escape from antitumor immunity, thereby leading to the development of diseases such as MM.

Our previous study reported that CD8^+^ lymphocytes of patients with MM showed a decrease in intracellular perforin levels after stimulation and tended towards lower IFN-*γ* production compared to HV or patients with PP [16]. The present study examined functions of EBT-8-Org and EBT-8-CH30, and results showed that intracellular expression of perforin and production of IFN-*γ* were reduced in EBT-8-CH30, the subline exposed to long-term chrysotile. Thus, EBT-8-CH30 displayed several characteristics representing functional alterations, similar to that of peripheral blood CD8^+^ T cells in patients with mesothelioma. That similarity supports the idea that inhaled asbestos might chronically cause impaired function of CD8^+^ T cells and is linked to the development of MM in people exposed to asbestos. Although it cannot be denied that the characteristics of CD8^+^ T cells in patients might be influenced by immune-suppressive factors derived from the tumor, our present study clearly demonstrated that long-term exposure to asbestos specifically leads to the impairment of functions of CD8^+^ T cells. On the other hand, it remains to be clear how chronic exposure to chrysotile affected functions of CD8^+^ T cells. As chrysotile tends to have outer layer degrade in an aqueous environment for long period [23], it may be important to examine the effect of such “leached chrysotile” as well as native chrysotile fibers in the future study.

It is noteworthy that chronic exposure to chrysotile resulted in a difference in expression of perforin and granzyme B in the present study and was manifested by a decrease in the number of perforin-positive cells, unlike the case with granzyme B MFI. Perforin is expressed in cytotoxic lymphocytes including NK, CD8^+^, and CD4^+^ T (Th1) cells, but is not expressed in other cell types including B cells and nonlymphoid cells [24, 25]. Additionally, human CD8^+^ T lymphocytes in peripheral blood as well as lymphoid organs do not express perforin at the protein level and do not show cytolytic activity prior to stimulation, and perforin protein is predominantly expressed in effector CD8^+^ T cells in humans [25–27]. In contrast to perforin, granzyme B is expressed in many types of human nonlymphoid cells including plasmacytoid, dendritic cells, and IL-3-stimulated basophils [28–30]. Those findings suggest that perforin expression might be more strictly controlled and may be more crucial in contributing towards the cytotoxic role of CD8^+^ T lymphocytes compared to granzyme B, which might explain the difference in expression of perforin and granzyme B determined in our present study as mentioned above. Alternatively, the redundancy of granzyme genes may be important in considering these results. Five granzyme genes are known in humans, granzyme A, B, H, K, and M, while ten granzymes have been identified in mice [31]. As our present study only examined the expression of granzyme B, it is possible that expression of the other granzymes might be altered by chronic exposure to chrysotile asbestos. Additionally, a deficiency in one of the ten granzymes in mice led to only subtle alterations in antivirus and antitumor immunity, while a deficiency in perforin caused severe immunodeficient characteristics [31]. Those experiments perhaps underline the greater importance of perforin in CTL functions compared to granzymes and which may be reflected in the difference in perforin and granzyme expression under chronic exposure to chrysotile. There is actually a difference in the MFI of granzyme B between EBT-8-Org shown in Figures 2 and 3. This difference is probably derived from the altered function of EBT-8 cells before the start of the culture with chrysotile. We know from our own experience that EBT-8 cells show alterations in expression of perforin and granzyme B depending on the those conditions, which includes cell number in cryotubes, and the length of time cells have been stored frozen. Therefore, our present study undertook repeated examination of EBT-8 cells (*n* = 14 in each of Figures 2 and 3) and demonstrated significant differences between control and chrysotile-exposed cultures, as shown in Figures 2 and 3.

The secreted production of IFN-*γ* stimulated via CD3 decreased with CH exposure, whereas the percentage of IFN-*γ*^+^ cells induced by PMA/ionomycin did not decrease. We previously examined the percentage of cells positive for intracellular IFN-*γ* in CD8^+^ lymphocytes involved in peripheral blood mononuclear cells (PBMCs) harvested after 7 days of a mixed lymphocyte reaction (MLR) in the presence of 5 *μ*g/ml of CH asbestos, where PMA/ionomycin was used to stimulate those harvested cells for 4 h prior to the assay for intracellular IFN-*γ* [32]. In that study, it was confirmed that asbestos exposure suppressed the differentiation of human mature CTLs during the MLR and was accompanied by decreases in the percentage of IFN-*γ* in CD8^+^ lymphocytes. Additionally, we previously confirmed a trend showing decreased percentage of cells positive for intracellular IFN-*γ* in CD8^+^ lymphocytes from PMA/ionomycin-stimulated PBMCs derived from patients with MM [16]. Therefore, it was our first choice to use PMA/ionomycin as a stimulator to examine the percentage of IFN-*γ*^+^ cells in EBT-8-CH5 and EBT-8-Org. However, there was no difference in the percentage of IFN-*γ*^+^ cells between EBT-8-CH5 and EBT-8-Org. Then, we used bead-bound anti-CD3 Abs, to represent greater physiological stimulation, for 48 hours and examined the effect of 30 *μ*g/ml asbestos on the secretory production of IFN-*γ*. These results showed suppressed production of IFN-*γ* by EBT-8-CH30, the subline which suffered from long-term exposure to CH asbestos. These findings suggest that lasting exposure to asbestos might impair production of IFN-*γ* by influencing the machinery of T cell stimulation with CD3. On the other hand, there was no difference in the expression of CD107a, a marker for degranulation, after stimulation with CD3 between EBT-8-Org and EBT-8-CH30 sublines. This finding suggests partial, but not total, impairment of the signal transduction machinery within T cells stimulated with CD3 and may provide a valuable key in efforts to explore the mechanism of signal transduction impairment in EBT-8-CH30. Additionally, it cannot be ignored that the different results between percentage of cells positive for (Figures 5(a) and 5(b)) and secreted amount of IFN-*γ* (Figure 5(c)) might be attributed to the difference between EBT-8-CH5 and EBT-8-CH30. However, as both of the cell lines showed the asbestos exposure-caused effect on perforin expression, it may be more probable that the difference between Figures 5(a)–5(c) is related to the difference in pathways of stimulation.

## 5. Conclusion

Our present investigation is the first to show that *in vitro* long-term exposure to asbestos has the potential to suppress certain functions of CD8^+^ T cells. It is known that tumors cause immune-suppressive environments [33]. However, the findings obtained from the present study demonstrated that long-term exposure to asbestos impairs the performance of CD8^+^ T cells alone without any factors derived from tumors. Additionally, by using an *in vitro* model to analyze the effect of chronic exposure to inhaled asbestos, our present study demonstrated that low- and middle-dose asbestos exposure suppresses functions of CD8^+^ T cells. An additional study using another cell line in future might contribute to confirmation of the present results. Further investigations are needed to clarify the mechanisms involved in the suppression of CD8^+^ T cell functions upon exposure to asbestos, with the ultimate goal being to prevent the development of MM and other tumors in people who have inhaled asbestos.

## Data Availability

The data used to support the findings of this study are available from the corresponding author upon reasonable request, only if could obtain permission of the data available from Dr. Asada, because the present study was accomplished by using EBT-8, which was given kindly by him, who had originally established the cell line, under the agreement about the usage of it between each other.
